# Trouble always comes in threes: three mutations for three auto inflammatory genes in a child and in his father

**DOI:** 10.1186/1546-0096-12-S1-P265

**Published:** 2014-09-17

**Authors:** Maria Cristina Maggio, Carmelo Fabiano, Eugenia Prinzi, Giovanni Corsello

**Affiliations:** 1 Pro.Sa.M.I. "G. D'Alessandro", University of Palermo, Palermo, Italy; 2 Molecular Genetic Service, Ospedali Riuniti Villa Sofia – Cervello, Palermo, Italy

## Introduction

The coexistence of mutations in more than one gene, linked to Autoinflammatory Diesases, can confuse and make difficult the diagnosis and management of these patients, especially in childhood, when the clinical history is still brief.

## Objectives

However therapeutic decisions are often necessary and can significantly modify clinical outcome and long term prognosis.

## Methods

We report the clinical case of a 4 years old child who presented arthralgia since the age of 2,5 years. An orthopaedic diagnosed them as “growing pain”. Some months later he presented fever, neutrophilic leucocytosis, significant elevation of CRP and ESR associated to legs pain; he was treated with antibiotics and NSAID with a poor response.

In the last year these episodes were monthly recurrent, associated with abdominal pain, oral aphtae, exanthema, conjunctivitis. The attacks are 3-4 sometimes 7 days long, have a monthly recurrence and do not show a seasonal prevalence. He showed a weight loss (weight: -2SDS; height: -1SDS); in the free intervals he was in wellbeing.

## Results

During an attack he showed: increased CRP, ESR, alfa2, IgA; neutrophilic leucocytosis, serum amyloid-A (SAA): 65 mg/L (n.v. <6). In the free- attack period the SAA was in the normal range.

The genetic study of FMF, TRAPS, MVK evidenced the following mutations: heterozygous mutation of MEFV gene: K695R; heterozygous mutation of TNFRS1A gene: P46L. He started colchicine with only a moderate improvement of clinical and laboratory data. He prolonghed this treatment for three months but for the persistence of symptoms and elevated SAA levels a treatment with biologic drugs anti-IL1 was programmed.

The genetic study was extended to the entire family: the father, a patient with Multiple Sclerosis (MS) diagnosed at 29 years, showed the same mutation of the son. The mother and the brother were negative. The father revealed a personal history of recurrence of pharyngitis, fever, arthralgia, legs myalgia, headhache, partially related also to MS.

The association between MEFV gene mutations and MS, the TNFRSF1A gene R92Q mutation and MS, in patients who refer symptoms related to TRAPS.

However a link between NLRP3 gene mutations and MS is also reported in patients in whom MR documented a partial reduction of lesions after anti-IL1 treatment. In these cases the anti-IL1 drugs remitted recurrent episodes related to CAPS.

The NLRP3 gene study was performed and revealed the mutation Q703K both in the father and in the son. The treatment with Canakinumab was started and a resolution of recurrent attacks in the son and a reduction of legs pain in the father. The treatment for MS is however continued in the father.

## Conclusion

The follow up is still ongoing; the relieve of the NLRP3 mutation permitted the treatment with anti-IL1 otherwise difficult.

A MS precocious onset could be a selection criteria for NLRP3 gene mutations study.

## Disclosure of interest

None declared.

